# Attitude and behaviour of Dutch Otorhinolaryngologists to Evidence Based Medicine

**DOI:** 10.1371/journal.pone.0226743

**Published:** 2019-12-30

**Authors:** Maaike M. Rademaker, Adriana L. Smit, Marlous F. Kortekaas, Peter Paul G. van Benthem, Inge Stegeman

**Affiliations:** 1 Division of Surgical Specialties, Department of Otorhinolaryngology-Head and Neck surgery, University Medical Center Utrecht, Utrecht, The Netherlands; 2 UMC Utrecht Brain Center, Utrecht, The Netherlands; 3 Julius Centre for Health Sciences and Primary Care, University Medical Center Utrecht, Utrecht, The Netherlands; 4 Department of Otorhinolaryngology-Head and Neck surgery, Leiden University Medical Centre Leiden, Leiden, The Netherlands; University of Porto Faculty of Medicine, PORTUGAL

## Abstract

**Objective:**

The objective of this study was to assess the attitude and behaviour of Dutch ENT surgeons and ENT residents towards Evidence Based Medicine.

**Introduction:**

Evidence Based Medicine is the cornerstone of modern clinical care. It is considered of crucial importance for optimal patient care and health care quality. Practicing it requires positive attitude and behaviour. Little is known about the attitude and behaviour towards Evidence Based Medicine in otorhinolaryngology.

**Methods:**

We performed a web-based questionnaire among 607 Dutch Ear- Nose & Throat surgeons of whom 106 residents (cross-sectional study). The questionnaire consisted of 3 parts; (1) personal characteristics, (2) questions regarding Evidence Based Medicine attitude (McColl questionnaire, scale 0–100%) and (3) questions regarding Evidence Based Medicine behaviour (barriers and information seeking behaviour). Data were collected between March 26^th^ 2018 and June 1^st^ 2018.

**Results:**

The median score on the overall McColl questionnaire was 50 (IQR 35). The main barriers respondents experienced were time related. Limited time in the outpatient clinic was considered a more important barrier for residents to practice EBM compared to ENT surgeons. Respondents’ gut feeling and their own preference were identified as the main contributing factors in clinical decision making.

**Conclusion:**

In conclusion Dutch ENT surgeons have a moderate attitude on the McColl questionnaire. The main barriers to practice Evidence Based Medicine they experience are time related.

## Introduction

Evidence Based Medicine (EBM) is the foundation modern clinical care is built on.[1] In 1996 the current definition of EBM was defined by Sackett et al. as: “the conscientious, judicious and explicit use of best available evidence, integrating with clinical judgment and patient values to provide the best individual care for the patient”, which was based on philosophical ideas originating from mid-19^th^ century Paris.[2] EBM encourages clinicians to look at individual patients’ needs and to track down the best available evidence to answer individual clinical questions.[2]

The importance of EBM lies in its ambition to create optimal patient care. Literature reports that 73–84% of patients receive evidence based care.[3–5] McGlynn (2003) reported that 11% of patients received care not in accordance with the latest evidence and potentially harmful care.[6] Implementation of EBM can be achieved by spreading the outcomes of clinical studies in clinical journals, at conferences, and by the creation of evidence based guidelines [7] Nonetheless, a survey assessing guideline adherence in Otorhinolaryngology showed a nonadherence of 45% to guidelines, most probably due to guidelines that do not provide strict recommendations [8]. Even with improving modern techniques to disseminate evidence through the internet (UpToDate, PIER, Clinical Evidence), implementation in clinical practice continues to be difficult.[9]

To overcome the disagreement between science and practice, Evidence Based Practice (EBP) was developed.[10] By adhering to 5 steps -*Ask*, *Access*, *Appraise*, *Apply and Assess*, a physician is assisted in integrating scientific evidence into daily practice.[11] It is estimated that 2 questions are raised for every 3 patients a surgeon sees.[12,13] However, surgeons only search for answers in 50% of the questions.[13] We believe this might be caused by barriers experienced by surgeons to practice EBM[14] Barriers differ among different types of health care providers, e.g. general practitioners versus secondary care or consultants versus residents.[9,15,16]

Practicing Evidence Based Medicine requires specific competencies including knowledge and skills. Besides, individual attitude and behaviour towards EBM are of prime importance to properly practice EBM.[17] Research about the knowledge of, skills in, and attitude and behaviour towards EBM was performed in several medical fields.[15,18–20] To the best of our knowledge no research of this kind was performed in the field of otorhinolaryngology. Therefore, to gain insight in possible improvements in EBM adherence, we assessed the attitude and behaviour of Dutch Ear- Nose & Throat (ENT) surgeons towards EBM.

## Methods

### Study population

All currently practicing ENT surgeons (n = 501) and ENT residents (n = 106) in the Netherlands, who were registered as a member of the Dutch Society of Otorhinolaryngology—Head and Neck Surgery (Nederlandse Vereniging voor Keel-Neus-Oorheelkunde en Heelkunde van het Hoofd-Halsgebied) at 26-03-2018, were included. There were no exclusion criteria. Informed consent was considered provided if a participant filled out the questionnaire. General data on Dutch Otorhinolaryngologists were provided by the Dutch Society of Otorhinolaryngology—Head and Neck Surgery, and extracted from their website at the 16^th^ of April 2018. The Medical Ethical Research Committee of the University Medical Centre Utrecht (UMCU) judged that the Medical Research Involving Human Subjects Act does not apply for the study (February 28^th^ 2018).

### Questionnaires

The first part of the questionnaire consisted of three sections: (1) personal characteristics, (2) attitude towards EBM and (3) behaviour towards EBM. The complete questionnaire can be found in the supporting information.(S1 File Questionnaire English and S2 File Questionnaire Dutch)

1. Personal characteristics: i.e. sex, year of birth, year of registry within the database of Dutch Society of Otorhinolaryngology—Head and Neck Surgery, PhD fulfilment, and type of employment. A self-report question about EBM attitude was asked, using a 5-point Likert scale. 1: very unimportant, 5: very important.
2. EBM attitude. Attitude was defined as the mind-set of the responders as to the principles of EBM.[15] It was assessed using the validated McColl Questionnaire (1998) which consists of seven questions and which was forward-backward translated into Dutch.[15,21] One question was assessed with a scale ranging from 0% (very negative) to 100% (very positive). The other six questions were assessed with a scale ranging from 0% (very positive) to 100% (very negative). These scores were inverted prior to statistical analysis.
3. EBM behaviour. EBM behaviour was assessed in two ways. First, we investigated the barriers to apply EBM based on a validated questionnaire consisting of 19 statements.[19] The questionnaire assesses questions on a 5-point Likert scale (1 = totally disagree, 5 = totally agree). The original validated questionnaire was adjusted. One question was removed as we considered it irrelevant to the field of otorhinolaryngology. If necessary statements were minimally adjusted to adapt to the field of otorhinolaryngology. For one question (*Q17 my residents and interns motivate me to work according to EBM*) one option was added to the scale (6 = not applicable).

In the second part of the questionnaire we examined information seeking behaviour, based on a not-validated Dutch questionnaire that was developed for general practitioner trainees (unpublished data). This questionnaire consisted of 7 questions encompassing (1) access and usage of scientific information and (2) factors contributing to clinical decision making. If necessary questions were minimally adjusted to fit otorhinolaryngology.

### Logistics

A questionnaire was distributed at the 27^th^ of March 2018 to the members by the Dutch society of Otolaryngology—Head and Neck surgery through an email alert. Information on the study and an URL link to the questionnaire were provided in the email. The questionnaire was administered in an electronic questionnaire system: NetQuestionnaires. To maximize response rate, several actions were taken. First, the Dutch society of Otolaryngology- Head and Neck surgery sent a reminder email 2 weeks after the initial email. Second, a reminder email was sent after 4 weeks directly to ENT surgeons and residents. Third, to increase awareness, a reminder was added to the PowerPoint presentation of 1 colleague of the otorhinolaryngology department of the UMCU, at the biannual congress for Dutch ENT surgeons. Respondents were able to fill out the questionnaire till the 1st of June 2018.

### Outcomes

Primary outcomes were EBM attitude and behaviour. The secondary outcome was information seeking behaviour. The outcomes were measured using the questionnaires as described under methods.

### Statistical analysis

After the questionnaires were completed, the answers were automatically saved in NetQuestionnaires. Data of completed questionnaires were exported to an SPSS file. All data were analysed in SPSS version 21.0. We visually checked data for normality and performed Kolmogorov-Smirnov and Shapiro-Wilk tests of normality. Normally distributed data was presented as means with standard deviations. For not normally distributed data medians and quartiles were calculated. Mann-Whitney U tests were used to compare different groups. Chi-square tests were used to compare difference is categorical data. For question 2, part 3, the average was analysed, if participants answered with more than one number.

## Results

Of the 501 ENT surgeons and 106 ENT residents, 103 (17%) respondents started the questionnaire. 58 (12%) ENT surgeons and 10 residents (9%) completed the questionnaire (total n = 68, 11%), only data from respondents that completed the questionnaire was analysed. Characteristics of (non)responders (sex, year of birth, registry time and type of employment) are presented in Table 1. Baseline characteristics of responders and non-responders were similar (Table 1).

**Table 1:** Baseline characteristics.

Characteristics	Respondents			Dutch Otorhinolaryngologists*		
	ENT surgeons (n = 58)	ENT Residents (n = 10)	Total (n = 68)	ENT surgeons (n = 501)	ENT Residents (n = 106)	Total (n = 607)
Gender						
• Male	38 (66)	5 (50)	43 (63)	338 (67)	41 (39)	379 (62)
• Female	20 (34)	5 (50)	25 (37)	163 (33)	65 (61)	228 (38)
PhD	36 (62)	3 (30)	39 (57)	255 (51)	27 (25)	282 (46)
Year of Birth						
• 1998–1989	0 (0)	2 (20)	2 (3)	0 (0)	24 (23)	24 (4)
• 1988–1979	23 (40)	8 (80)	31(46)	122 (24)	81 (76)	203 (33)
• 1978–1969	14 (24)	0 (0)	14 (21)	167 (33)	1 (1)	168 (28)
• 1968–1959	16 (28)	0 (0)	16 (24)	136 (27)	0 (0)	136 (22)
• ≤ 1958	5 (9)	0 (0)	5 (7)	76 (15)	0 (0)	76 (13)
Registry time, y						
• < 10 (2008–2017)	32 (55)			210 (42)		
• 11–20 (1998–2007)	14 (24)			157 (31)		
• 21–30 (1988–1997)	10 (17)			109 (22)		
• > 30 (< 1987)	2 (3)			25 (5)		
Employment in type of workplace°						
• Academic	21 (33)			132 (25)	106 (100)	238 (38)
• General	40 (64)			368 (70)		
• Private Clinics	2 (3)			26 (5)		

Data is noted as number (percentage) °Employment in type of workplace is defined as total number of employments (responding ENT surgeons n = 63 and ENT surgeons, data from society n = 526 (16-4-2018)) * General data on Dutch Otorhinolaryngologists were provided by the Dutch Society of Otorhinolaryngology—Head and Neck Surgery, and extracted from their website at the 16^th^ of April 2018.

### EBM attitude

The overall median of the McColl questionnaire was 50 (Interquartile range (IQR) 35) (Table 2). The outcome of the self-rating attitude question towards EBM was high (median 4, IQR 0, on a 5 point-Likert scale, 1: very unimportant, 5: very important). We found no significant differences in the single self-reported attitude when comparing (1) ENT residents to ENT surgeons and (2) ENT surgeons with different registry time. However, comparing the outcome of the McColl questionnaire, one significant difference (p = 0.023) was found in question 2: ‘How would you describe the attitude of most of your colleagues towards EBM’ between residents (median 30, IQR 12) and ENT surgeons (median 51, IQR 33). No significant differences were found between ENT surgeons with different registry time in the McColl questionnaire.

**Table 2:** Scores on the McColl questionnaire.

	Residents (n = 10)	ENT surgeons (n = 58)	P =	All respondents (n = 68)
1. How would you describe your own attitude towards the current promotion of EBM?	31 (33)	50 (44)	0.123	49 (43)
2. How would you describe the attitude of most of your colleagues towards EBM?	30 (12)	51 (33)	**0.023**	50 (32)
3. How useful is EBM in your day-to-day management of patients?	35 (42)	54 (43)	0.182	53 (45)
4. What percentage of your clinical practice is currently evidence based?	70 (41)	52 (27)	0.260	53 (28)
5. Practicing EBM improves patient care	25 (66)	50 (58)	0.246	46 (60)
6. EBM is of limited value in otorhinolaryngology because much of patient care lacks scientific base	63 (54)	52 (48)	0.856	54 (47)
7. The adoption of EBM, however worthwhile as an ideal, places another demand on the already overloaded ENT surgeon.	52 (42)	40 (58)	0.182	41 (57)

Presented as median (IQR) IQR = Interquartile Range. Bold numbers indicate statistical significance (p < 0.05)

### EBM behaviour

#### Barriers

The most important barriers for EBM were: *when busy*, *searching for clinical evidence is not a priority to me* (median 4, IQR 2), and *the time I have per patient is insufficient to also search for answers to my questions (according to the principles of EBM)* (median 4, IQR 1) (Table 3). For the question: ‘During consultations, I have sufficient time to work according to the principles of EBM’, a significant difference (0.047) was found between ENT residents (median 2, IQR 1) and ENT surgeons (median 2.5, IQR 2). We found no significant differences when comparing ENT surgeons with different registry time.

**Table 3:** Barriers to practice EBM.

Barrier	Residents (n = 10)	ENT surgeons (n = 58)	P =	All respondents (n = 68)
As a result of inexperience with one (or more) of the EBM steps, I do not succeed at using EBM in practice	2 (1)	2 (1)	0.821	2 (1)
As a result of a lack in education in using EBM, I am unsure of what using EBM practically means	2 (0)	2 (2)	0.743	2 (2)
During outpatient clinic consultations, I have sufficient time to work according to the principles of EBM	2 (1)	2.5 (2)	**0.047**	2 (2)
When I search for evidence I do not know when to be pleased with the answer found	2 (1)	2 (2)	0.378	2 (2)
I appreciate it when colleagues present me with new evidence	4 (0)	4 (1)	0.094	4 (1)
I am not interested in searching for the best evidence	2 (1)	2 (1)	0.498	2 (1)
To answer a clinical question I prefer a quick method over a precise method	3.5 (2)	3 (2)	0.693	3 (2)
Searching for clinical evidence is hard for me	2 (2)	3 (2)	0.401	3 (2)
I don’t search for clinical evidence because I trust the national ENT guidelines	3 (1)	3 (2)	0.767	3 (2)
When busy, searching for clinical evidence is not a priority to me	3 (2)	4 (1)	0.249	4 (2)
My skills in searching for evidence in databases (i.e. Pubmed) are sufficient	4 (2)	4 (1)	0.741	4 (1)
The critical appraisal of literature is not hard for me	3.5 (2)	3 (1)	0.497	3 (1)
When I have a clinical question, I take the initiative to search for an evidence-based answer	4 (1)	4 (1)	0.569	4 (1)
My colleagues (otorhinolaryngologists) stimulate me in practicing EBM	3 (1)	3 (1)	0.882	3 (1)
I find the articles written in English difficult	1 (1)	1 (1)	0.984	1 (1)
I am not motivated in working according to the principles of EBM	1.5 (1)	2 (1)	0.251	2 (1)
The time I have per patient is insufficient to also search for answers to my questions (according to the principles of EBM)	4 (2)	4 (1)	0.903	4 (1)
My residents and interns motivate me to work according to the principles of EBM*	4 (2)	4 (2)	0.929	4 (2)

Scores are presented as median (IQR) IQR = Interquartile range. Items were assessed on a 5 point Likert scale (1: totally disagree, 5: totally agree.)

N = 68. Bold numbers indicate statistical significance (p < 0.05)

* a sixth option was added: not applicable

### Information seeking behaviour

Ninety percent of respondents performed or let someone perform literature searches in the last month before filling out the questionnaire; these respondents performed a median of four literature searches (median, IQR 4). This search influenced clinical practice in half of the times (median 50, IQR 43). Of all respondents, 74% had some form of EBM training.

**Fig 1:**
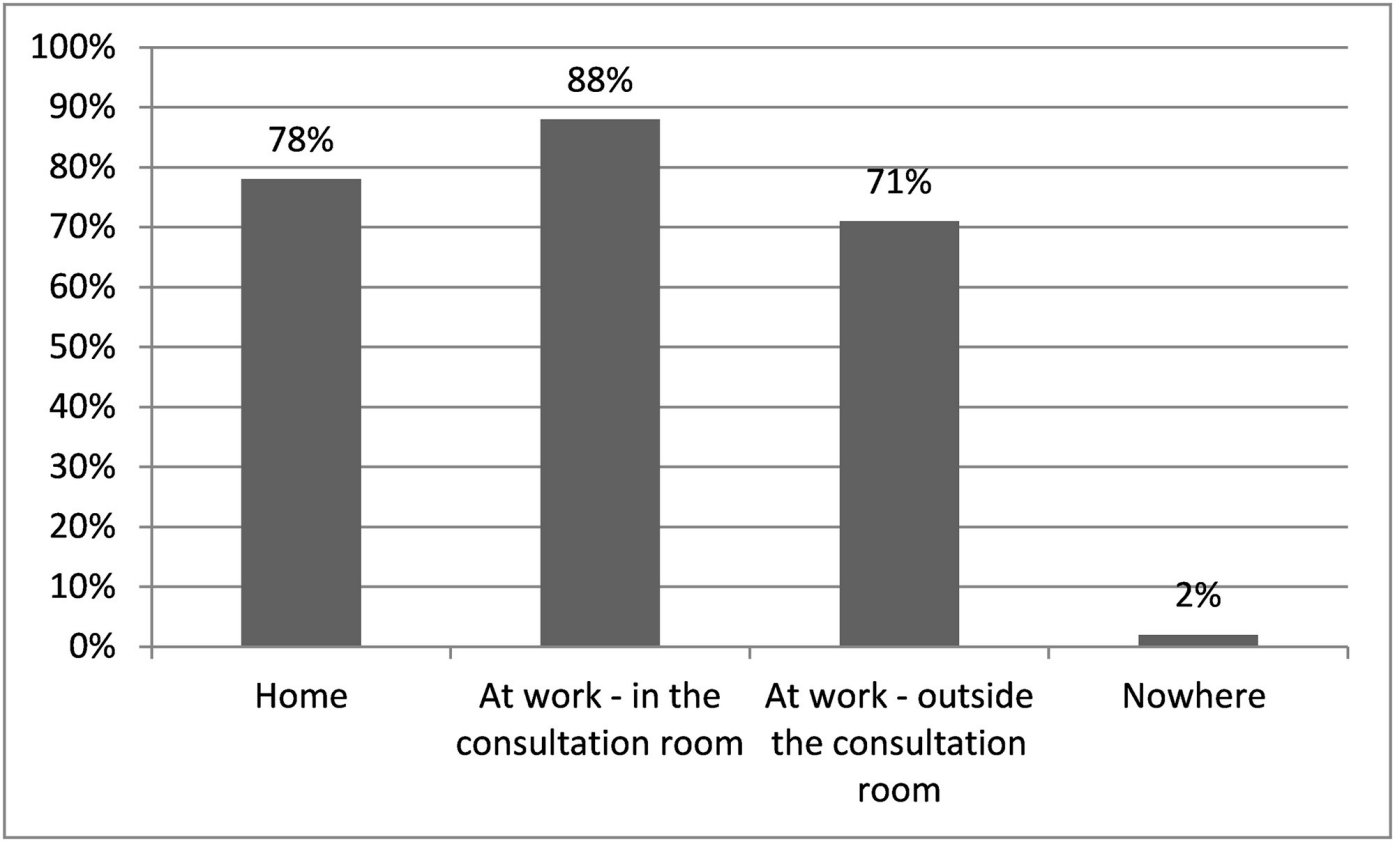
Access to full-text files from different databases by all respondents (n = 68).

88% of respondents have access to full-text articles at work—in the consultation room (Fig 1). No significant differences were found between residents and ENT surgeons, or within ENT surgeons with different registry time.

**Fig 2:**
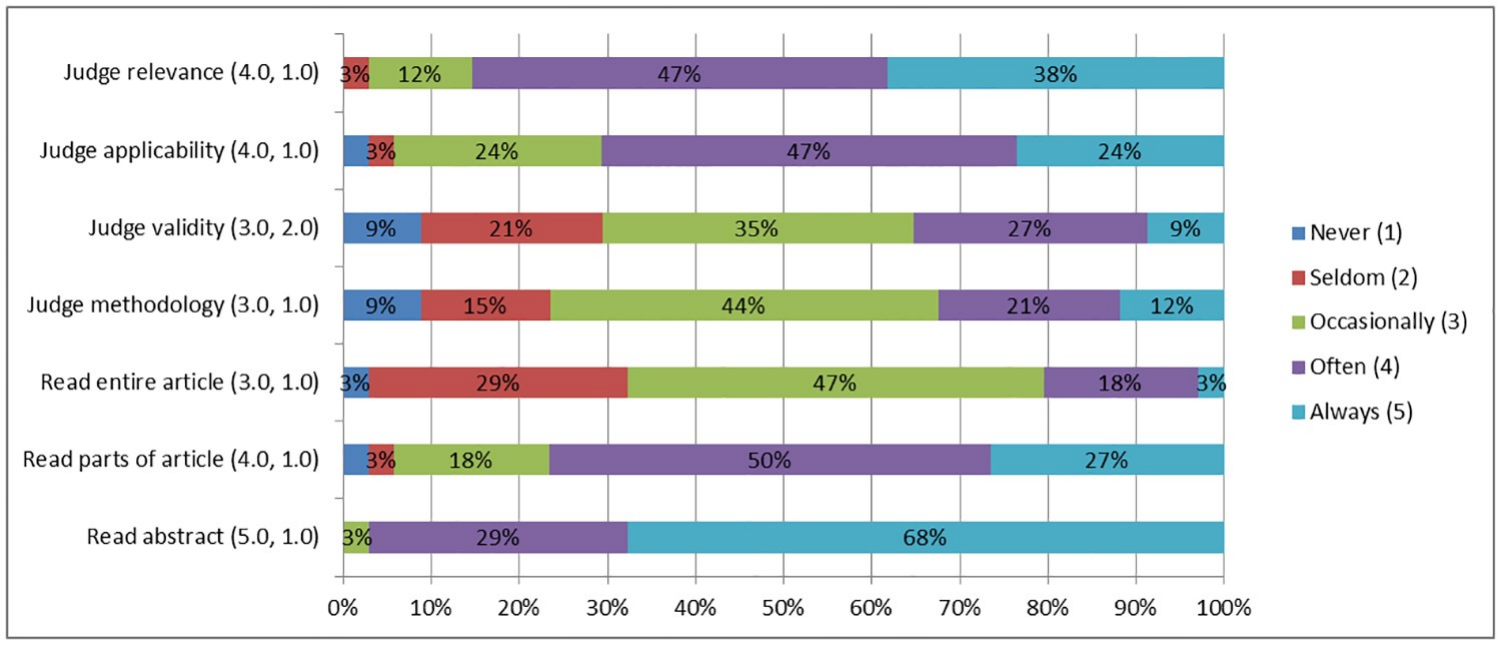
Reading- (portion) and judgement of articles read in the past two weeks (n = 34). Data is pictured as: (median, interquartile range).

Half of respondents had performed a literature search in the two weeks before participating in the questionnaire. Of those, 50% often read parts of the article (median 4.0, IQR 1.0), while 3% always read the entire article (median 3.0, IQR 1.0) (Fig 2). No differences were found between residents and ENT surgeons, and between ENT surgeons with different registry time.

**Fig 3:**
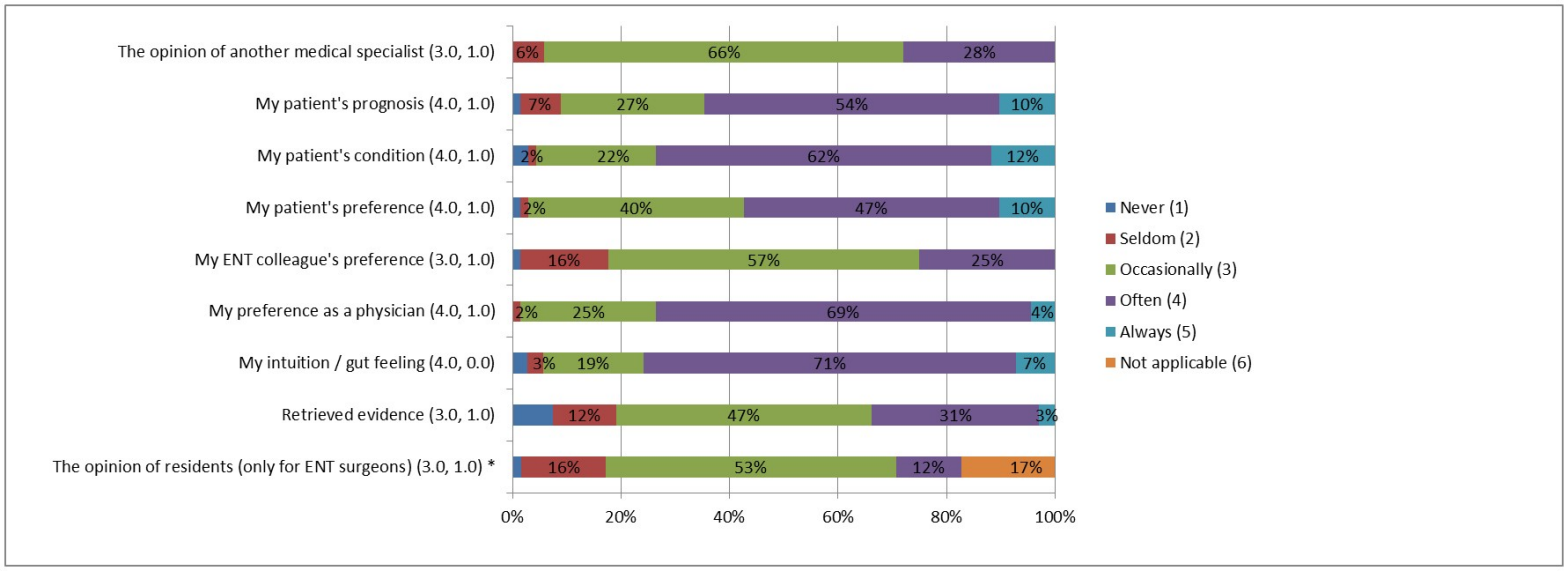
Factors contributing to clinical decisions (n = 68). Data is pictured as: (median, interquartile range),* only for ENT surgeons (n = 58).

Reported factors influencing clinical decision-making were diverse. The most important factors were the respondents’ own preference (median 4.0, IQR 1.0), the patient’s prognosis (median 4.0, IQR 1.0), the patient’s condition (median 4.0, IQR 1.0), the patient’s preference (median 4.0, IQR 1.0), and the ENT surgeons’ gut feeling (median 4.0, IQR 0.0) (Fig 3). A significant difference was found between ENT residents (median 4.0, IQR 1.0) and ENT surgeons (3.0, 0.0) in the factor *‘my colleague’s preference’* (p = 0.022). No significant differences were found between ENT surgeons with different registry time.

National Guidelines and PubMed/Embase were used often by most surgeons (Table 4). No significant differences were found between residents and ENT surgeons. ENT surgeons registered < 10 years used Pubmed / Embase more often (median 6, IQR 1) than their older colleagues registered > 30 years (median 4, IQR 0) (p = 0.043). ENT surgeons registered < 10 years used UpToDate (median 4,0, IQR 2) more often than their colleagues registered 20–30 years (median 3, IQR 3) (p = 0.03).

**Table 4:** Awareness and use of different databases by respondents (n = 68).

Database	Median	IQR
Magazine of (Dutch society of) otorhinolaryngology	5	2
Dutch Medical Magazine (NTvG)	4	2
Pubmed/Embase	6	1
National guidelines (ENT / CBO)	6	0
Google	5	2
Cochrane	4	1
UpToDate	4	2

1: unknown, 2: aware of the existence but I don’t use it, 3: I know how to find it but I don’t use it, 4: seldom use (<1x month), 5: occasional use (± 1 month), 6: often (weekly), 7: always (daily).

## Discussion

In this study, we investigated the attitude and behaviour of Dutch ENT surgeons towards EBM. We noticed an overall moderately positive attitude towards EBM. We identified several barriers for practicing EBM, with limited time as main barrier. Limited time in the outpatient clinic is a more important barrier for residents to practice EBM compared to ENT surgeons. By evaluating information seeking behaviour we identified the respondents’ own preference and gut feeling as the main contributing factors in their clinical decision making.

Even though working according to the principles of EBM (self-rated attitude) was considered ‘important’, the attitude towards EBM by the McColl as a validated multi-item questionnaire turned out to be moderate. The scores on the McColl test (median 50) are comparable to the outcome of a survey in Dutch general practitioners (mean 56–62.8) and Dutch general surgeons (individual answers ranging between 44 and 78).[15,19,22]

Several papers compare the mean McColl score to a single self-reported attitude and find significant overestimation of self-reported EBM attitude compared to the outcome of the McColl questionnaire.[22,23] We believe however, that the variety in questions of McColl is too high to justify direct comparison with self-reported attitude. It is interesting to compare self-reported attitude (median 4.0, important) to McColl question 4 (*what percentage of your clinical practice is currently evidence base*; median 53). This indicates that respondents understand the importance of EBM, but do not always practice it.

In accordance with literature we found that limited time is an important barrier to practice EBM. In clinical care time is scarce. [9,14,15] Dutch medical specialists spend 40% of their time on administration.[24] This administrative pressure might be of influence on the largest barrier: limited time. If the administrative load would be reduced, this ‘new extra’ time might be spent on practicing EBM. Residents score a statistically significant higher median score on the question: *During outpatient clinic consultations*, *I have sufficient time to work according to the principles of EBM* than ENT surgeons. This might be explained by factors related to their experience, such as clinical decision making. However we cannot confirm this in our data.

We found that access to full text-files from different databases differs between various locations. Access to full-text files also depends on the subscriptions to scientific magazines of the different hospitals or individuals. This, again, underlines the importance of the need for a movement towards open access publication.

Pubmed / Embase and ENT guidelines are the most popular databases used by the participants of our study. However, an earlier study in the Netherlands, showed that 45% of all Dutch ENT surgeons showed nonadherence to these guidelines.[8] Even though respondents consider the national guidelines as a popular database based on our study, they do not always adhere to their advices. This fits the outcome of our study of how ENT surgeons make clinical decisions in which ‘retrieving evidence’ scored relatively low compared to e.g. ‘gut feeling’ or ‘personal preferences.’

Some methodological issues need to be addressed. The response rate of only 11% is a limitation to our study. This might mean that some differences might not have been verified, due to reduced statistical power. The recruitment through e-mail instead of postal services might be related to the limited response rate. Also the electronic questionnaire system was technically not accessible for mobile Apple products. Another explanation might be that the respondents are saturated by the amount of questionnaires they might receive. One could argue that response bias is suspected by the differences in characteristics of the respondents. However as seen in Table 1, our study participants are representative of the complete population of Dutch ENT surgeons.

ENT surgeons registered less than ten years are over represented in our study. This might indicate a more positive attitude towards EBM, compared to ENT surgeons registered longer. ‘Younger’ surgeons might have had more experience with EBM in university and during their residency, because of the creation of EBM’s definition in 1996.[2] Surprisingly, our results do not show many differences in attitude and behaviour between groups of ENT surgeons with different decades of registration. This raises questions to whether the ‘extra’ education younger surgeons received are of major influence on EBP, or that older surgeons have actively retrained themselves. Another question raised is whether the attitude and behaviour on EBM is as important a factor on the actual practice of EBM, assuming that EBM attitude and behaviour have improved over the years. According to Chapman et al. there is little change in the amount of patients that receive evidence-based care in internal medicine, comparing data from 1995 and 2013. In 1995, 82% of internal medicine patients received evidence-based treatment, compared to 84% in 2013 [3,25]. To fully comprehend the extend of the influence of EBM attitude and behaviour in the Dutch ENT care; one first needs to perform an audit on the amount of evidence based care in Dutch ENT care. To our knowledge no study to this extend has been performed.

Future research should investigate how to solve the experienced barriers and the effect on practicing EBM. EBM competency is not limited to attitude and behaviour, but does also entail knowledge and skill.[17] In order to fully comprehend EBM competency in Dutch ENT surgeons, research assessing knowledge and skill would be additive to our current study.[26] Also, an educational intervention (face-to-face meetings, clinically integrated teaching) could improve EBM attitude and thereby indirectly influence EBM behaviour.[27,28]

In conclusion, Dutch ENT surgeons and residents scored moderately positive on the McColl questionnaire, assessing attitude. The main barriers they experience are time related. ENT surgeons use their own preference and gut-feeling most in making a clinical decision.

## Supporting information

S1 File Questionnaire. (DOCX)

S2 File Questionnaire. (DOCX)

S1 STROBE STROBE 2007 (v4) statement—Checklist of items that should be included in reports of *cross-sectional studies*. (DOCX)

S1 Dataset (SAV)

## References

[1] Kortekaas MF. Improving evidence-based general practice [Internet]. 2016. http://www.narcis.nl/publication/RecordID/oai%3Adspace.library.uu.nl%3A1874%2F338766

[2] Sackett DL, Rosenberg WMC, Gray J a M, Haynes RB, Richardson WS. Evidence based medicine: what it is and what it isn’t. BMJ [Internet]. 1996;312(7023):71–2. Available from: http://www.ncbi.nlm.nih.gov/pmc/articles/PMC2349778/pdf/bmj00524-0009.pdf 8555924 10.1136/bmj.312.7023.71 PMC2349778

[3] Chapman G, Talbot N, Mccartney D, Tippett V, Burch D. Evidence based medicine—older, but no better educated? in India faces new. Lancet [Internet]. 2013;382(9903):1484 Available from: 10.1016/S0140-6736(13)62286-2 24182541

[4] Ayre S, Mclip MA, Mrcp GW. Are therapeutic decisions made on the medical admissions unit any more evidence-based than they used to be? 2009;15:1180–6. 10.1111/j.1365-2753.2009.01345.x 20367724

[5] Asplund K. Randomized controlled trials and consensus as a basis for interventions in internal medicine. J Intern Med. 2000;247:94–104. 10.1046/j.1365-2796.2000.00583.x 10672136

[6] McGlynn EA, Asch SM, Adams JL, Keesey J, Hicks J, DeCristofaro AH, et al Quality of health care delivered to adults in the United States. N Engl J Med [Internet]. 2003;348:2635–45. Available from: http://www.ncbi.nlm.nih.gov/pubmed/14606462 12826639 10.1056/NEJMsa022615

[7] Grol R, Grimshaw J. From best evidence to best practice: Effective implementation of change in patients’ care. Lancet. 2003;362(9391):1225–30. 10.1016/S0140-6736(03)14546-1 14568747

[8] Aarts M.C.J., van der Heiden G.J.M.G., Siegers C. RMM Grolman W.. Awareness of, Opinions About, and Adherence to Evidence-Based Guidelines in Otorhinolaryngology. Arch Otorhinolaryngol Head Neck Surg. 2012;138(2):148–52. 10.1001/archoto.2011.1166 22351860

[9] van Dijk Nynke, Hooft Lotty de W MW. What are the barriers to residents´practicing evidence-based medicine? A systematic review. Acad Med. 2010;85(7):1163–70. 10.1097/ACM.0b013e3181d4152f 20186032

[10] Fineout-Overholt E, Melnyk BM, Schultz A. Transforming health care from the inside out: Advancing evidence-based practice in the 21st century. J Prof Nurs. 2005;21(6):335–44. 10.1016/j.profnurs.2005.10.005 16311228

[11] Straus S.E., Richardson W.S. G P. Evidence-based medicine, how to practice and teach EBM. 3rd ed Edinburgh: Churchill Livingstone; 2005.

[12] Covell D.G., Uman G.C. M PR. Information needs in office practice: are they being met? Ann Intern Med. 1985;103(4):596–9. 10.7326/0003-4819-103-4-596 4037559

[13] Del Fiol G, Workman TE, Gorman PN. Clinical questions raised by clinicians at the point of care a systematic review. JAMA Intern Med. 2014;174(5):710–8. 10.1001/jamainternmed.2014.368 24663331

[14] Zwolsman S, Hooft L, Waard MW, Van Dijk N. Barriers to GPs’ use of evidence-based medicine: A systematic review. Br J Gen Pract. 2012;62(600):e511–21. 10.3399/bjgp12X652382 22781999 PMC3381277

[15] Knops AM, Vermeulen H, Legemate DA, Ubbink DT. Attitudes, awareness, and barriers regarding evidence-based surgery among surgeons and surgical nurses. World J Surg. 2009;33(7):1348–55. 10.1007/s00268-009-0020-8 19412569 PMC2691930

[16] Funk SG, Champagne MT, Wiese RA, Tornquist EM. BARRIERS: The Barriers to Research Utilization Scale. Appl Nurs Res. 1991;4(1):39–45. 10.1016/s0897-1897(05)80052-7 1741634

[17] Dawes M, Summerskill W, Glasziou P, Cartabellotta A, Martin J, Hopayian K, et al Sicily statement on evidence-based practice. BMC Med Educ. 2005;5:1–7. 10.1186/1472-6920-5-1 15634359 PMC544887

[18] Kortekaas MF, Bartelink MEL, Van der Heijden GJMG, Hoes AW DW N. Development and validation of a new instrument measuring EBM behaviour in clinical practice. Submitted. 2016;33(2):1–7. 10.1093/fampra/cmw063 27461491

[19] Zwolsman SE, van Dijk N, Te Pas E, Wieringa-de Waard M. Barriers to the use of evidence-based medicine: knowledge and skills, attitude, and external factors. Perspect Med Educ [Internet]. 2013;2(1):4–13. Available from: http://www.ncbi.nlm.nih.gov/pubmed/23670651%5Cnhttp://www.pubmedcentral.nih.gov/articlerender.fcgi?artid=PMC3576485 23670651 10.1007/s40037-013-0039-2 PMC3576485

[20] Elbers NA, Chase R, Craig A, Guy L, Harris IA, Middleton JW, et al Health care professionals’ attitudes towards evidence-based medicine in the workers’ compensation setting: a cohort study. BMC Med Inform Decis Mak. 2017;17(1):1–12. 28532470 10.1186/s12911-017-0460-2 PMC5440905

[21] Mccoll A, Smith H, White P, Field J. Information in practice based medicine: a questionnaire survey. EvidenceBased Med [Internet]. 1998;316(February 2009):361–7. Available from: http://www.ncbi.nlm.nih.gov/pubmed/10674320 10.1136/bmj.316.7128.361 PMC2665572 9487174

[22] Te Pas E, Van Dijk N, Bartelink MEL, Wieringa-De Waard M. Factors influencing the EBM behaviour of GP trainers: A mixed method study. Med Teach. 2013;35(3):e990–7. 10.3109/0142159X.2012.733044 23102157

[23] Te Pas E, De Waard MW, De Ruijter W, Van Dijk N. Learning results of GP trainers in a blended learning course on EBM: A cohort study. BMC Med Educ. 2015;15(1). 10.1186/s12909-015-0386-2 PMC4472415 26067056

[24] VvAA en het Roer Moet OM. Administratiedruk medisch specialisten. De Arugmentenfabriek [Internet]. online pub. https://www.demedischspecialist.nl/sites/default/files/20171117_DEF%20Rapport-administratiedruk-specialisten.pdf

[25] Ellis J, Mulligan I, Rowe J, Sackett DL. Inpatient general medicine is evidence based. Lancet. 1995;346:407–10. 7623571

[26] Fishbein, M., & Ajzen I. Belief, Attitude, Intention, and Behavior: An Introduction to Theory and Research [Internet]. 1975 [cited 2018 May 15]. http://people.umass.edu/aizen/f&a1975.html

[27] Ireland J., Johnson N., Adams D., Eboh w. M E. Blended learning in education: effects on knowledge and attitude. Br J Nurs. 2009;18(2):124–30. 10.12968/bjon.2009.18.2.37868 19270612

[28] Coomarasamy A, Khan KS. What is the evidence that postgraduate teaching in evidence based medicine changes anything? A systematic review. BMJ. 2004;329(7473):1017 10.1136/bmj.329.7473.1017 15514348 PMC524555

