# Supplementary material for: Attitude and behaviour of Dutch Otorhinolaryngologists to Evidence Based Medicine
Source: PLoS One. 2019 Dec 30;14(12):e0226743. doi: 10.1371/journal.pone.0226743 (PMC6936769; doi:10.1371/journal.pone.0226743)
Supplement: S1 File — (DOCX) [file pone.0226743.s001.docx]

**Please note, the text in this document has been freely translated. The translation has not been validated.**

**Part 1**

1. What is your gender? Male/Female
2. Wat is your year of birth?
3. In which town did you attend medical school

- Groningen
- Amsterdam (Vrije Universiteit)
- Utrecht
- Nijmegen
- Amsterdam (Universiteit van Amsterdam)
- Leiden
- Maastricht
- Rotterdam
- Other, namely

1. In which town did you attend your training to become an ENT surgeon?

- Groningen
- Amsterdam (Vrije Universiteit)
- Utrecht
- Nijmegen
- Amsterdam (Universiteit van Amsterdam)
- Leiden
- Maastricht
- Rotterdam
- Other, namely

1. What year did you graduate as a doctor?
2. What year did you (will you) graduate as an ENT surgeon?
3. Do you have multiple scientific publications on your name as (co) author? Yes/No
4. Did you obtain a PhD? Yes/No
5. Select where you work. (more than one option)
   Peripheral hospital / Academic hospital / independent treatment centre
6. How many FTE do you work?
7. What do you think about you knowledge on Evidence-Based Medicine (EBM)?
   Moderate / average / good / very good
8. How important do you consider working according to the EBM principles?

Very unimportant / unimportant / neutral / important / very important

1. Do you supervise residents? Yes/No
2. Do you supervise interns? Yes/No

**Part 2**
the MCCOLL Questionnaire(1,2)

1. Mccoll A, Smith H, White P, et al. Information in practice based medicine : a questionnaire survey. *EvidenceBased Med*. 1998;316(February 2009):361–7.

2. Knops AM, Vermeulen H, Legemate DA, et al. Attitudes, awareness, and barriers regarding evidence-based surgery among surgeons and surgical nurses. *World J Surg*. 2009;33(7):1348–55.

**Part 3**Question 1 is based on the barriers questionnaire(3).

3. Zwolsman SE, van Dijk N, Te Pas E, et al. Barriers to the use of evidence-based medicine: knowledge and skills, attitude, and external factors. *Perspect Med Educ*. 2013;2(1):4–13.

Since several adjustments were made we present the questionnaire in the way we used in the survey. Please note that the original questionnaire was in Dutch.

|  | Totally disagree | Disagree | Neutral | Agree | Totally agree |
| --- | --- | --- | --- | --- | --- |
| 1. As a result of inexperience with one (or more) of the EBM steps, I do not succeed at using EBM in practice |  |  |  |  |  |
| 1. As a result of a lack in education in using EBM, I am unsure of what using EBM practically means |  |  |  |  |  |
| 1. I am not motivated in working according to the principles of EBM |  |  |  |  |  |
| 1. My skills in searching for evidence in databases (i.e. Pubmed) are sufficient |  |  |  |  |  |
| 1. I am not interested in searching for the best evidence |  |  |  |  |  |
| 1. Searching for clinical evidence is hard for me |  |  |  |  |  |
| 1. I don’t search for clinical evidence because I trust the national ENT guidelines |  |  |  |  |  |
| 1. I find the articles written in English difficult |  |  |  |  |  |
| 1. When I search for evidence I do not know when to be pleased with the answer found |  |  |  |  |  |
| 1. When I have a clinical question, I take the initiative to search for an evidence-based answer |  |  |  |  |  |
| 1. When busy, searching for clinical evidence is not a priority to me |  |  |  |  |  |
| 1. I appreciate it when colleagues present me with new evidence |  |  |  |  |  |
| 1. The critical appraisal of literature is not hard for me |  |  |  |  |  |
| 1. To answer a clinical question I prefer a quick method over a precise method |  |  |  |  |  |
| 1. During outpatient clinic consultations, I have sufficient time to work according to the principles of EBM |  |  |  |  |  |
| 1. The time I have per patient is insufficient to also search for answers to my questions (according to the principles of EBM) |  |  |  |  |  |
| 1. My colleagues (otorhinolaryngologists) stimulate me in practicing EBM |  |  |  |  |  |

|  | Totally disagree | Agree | Neutral | Agree | Totally agree | Not applicable |
| --- | --- | --- | --- | --- | --- | --- |
| 1. My residents and interns motivate me to work according to the principles of EBM |  |  |  |  |  |  |

2. a) how many times did you (or somebody for your) perform a search to scientific literature in the last month?

b) How many times did it influence your clinical work?

3. Have you ever had training on EBM?

4. Where do you have access to full-texts in Medline/Pubmed (or other bibliographical databases)?
- At home
- at work, in the consulting room
- at work, outside the consulting room
- somewhere else, namely:
- nowhere

5. There are beside the ENT guideline, a growing number of journals, review publications and databases that are relevant to EBM. Would you like to say in which manner you are familiar with these sources or use them (you can select multiple options)

| Source | Unknown | I am aware of the existence, but i do not use it | I know how to find it, but i do not use it. | I use it in clinical decision making | | | |
| --- | --- | --- | --- | --- | --- | --- | --- |
|  |  |  |  | Seldom (<1x/month) | Sometimes (± 1x/month) | Often  (weekly) | Always  (daily) |
|  | | | | | | | |
| ENT journal |  |  |  |  |  |  |  |
| NTvG (‘’Nederlands Tijdschrijft voor Geneeskunde’’) |  |  |  |  |  |  |  |
| Pubmed / Embase |  |  |  |  |  |  |  |
| Guidelines (ENT/CBO) |  |  |  |  |  |  |  |
| Google |  |  |  |  |  |  |  |
| Cochrane |  |  |  |  |  |  |  |
| Uptodate |  |  |  |  |  |  |  |

6. Did you look for original (primary) studies in the last two weeks? (if no please go to question 8) Yes / No

7. If you have sought for original (primary) studies, how extensively did you read and judge these?

|  | Never | Seldom | Sometimes | Often | Always |
| --- | --- | --- | --- | --- | --- |
| Read Abstract |  |  |  |  |  |
| Read parts of article |  |  |  |  |  |
| Read entire article |  |  |  |  |  |
| Judge methodology |  |  |  |  |  |
| Judge applicability |  |  |  |  |  |
| Judge Relevance |  |  |  |  |  |

8. How much did the coming factors contributed to you clinical decision making in the past two weeks?

|  | Never | Seldom | Sometimes | Often | Always |
| --- | --- | --- | --- | --- | --- |
| Retrieved evidence |  |  |  |  |  |
| My intuition / gut feeling |  |  |  |  |  |
| My preference as a physician |  |  |  |  |  |
| My ENT colleague’s preference |  |  |  |  |  |
| My patient’s preference |  |  |  |  |  |
| My patient’s condition |  |  |  |  |  |
| My patient’s prognosis |  |  |  |  |  |
| The opinion of another medical specialist |  |  |  |  |  |

|  | Never | Seldom | Sometimes | Often | Always | Not applicable |
| --- | --- | --- | --- | --- | --- | --- |
| The opinion of residents (only for ENT surgeons) |  |  |  |  |  |  |
