# Supplementary material for: Attitude and behaviour of Dutch Otorhinolaryngologists to Evidence Based Medicine
Source: PLoS One. 2019 Dec 30;14(12):e0226743. doi: 10.1371/journal.pone.0226743 (PMC6936769; doi:10.1371/journal.pone.0226743)
Supplement: S2 File — (DOCX) [file pone.0226743.s002.docx]

# Questionnaire

**Vaardigheden en Gedrag**

Met het invullen van deze vragenlijst ga ik ermee akkoord dat de gegevens van deze vragenlijst worden gebruikt voor wetenschappelijk onderzoek. De vragenlijst is geanonimiseerd en zal volledig anoniem worden verwerkt. Individuele resultaten worden zonder mijn toestemming nooit aan derden bekend gemaakt.

🡪 Akkoord/niet akkoord

Voordat u aan de vragenlijst begint, leggen wij hieronder graag uit wat wij binnen dit onderzoek verstaan onder "evidence-based medicine"

**Definitie:**

De definitie van evidence-based medicine is: het zorgvuldig, expliciet en oordeelkundig gebruik van het huidige beste bewijsmateriaal om beslissingen te nemen voor individuele patiënten. Dit impliceert het integreren van individuele klinische expertise met het beste externe bewijsmateriaal vanuit systematisch onderzoek en de voorkeuren, wensen en verwachtingen van de patiënt.

**Deel 1.**

1. Wat is uw geslacht? man / vrouw
2. Wat is uw geboorte jaar?
3. Waar heeft u de opleiding geneeskunde gevolgd?

- Groningen
- Amsterdam (Vrije Universiteit)
- Utrecht
- Nijmegen
- Amsterdam (Universiteit van Amsterdam)
- Leiden
- Maastricht
- Rotterdam
- Anders, namelijk:

1. Waar heeft u de opleiding tot KNO arts gevolgd?

- Groningen
- Amsterdam (Vrije Universiteit)
- Utrecht
- Nijmegen
- Amsterdam (Universiteit van Amsterdam)
- Leiden
- Maastricht
- Rotterdam
- Anders, namelijk:

1. Wat is het jaar van uw afstuderen als arts?
2. Wat is het (verwachtte) jaar van uw afstuderen als KNO-arts?
3. Heeft u een of meerdere wetenschappelijke Ja / Nee
   publicaties op uw naam als (mede-)auteur?
4. Bent u gepromoveerd? Ja / Nee
5. Kruis aan waar u werkt (meerdere aan te vinken) Perifeer ziekenhuis

Academisch ziekenhuis
 ZBC

1. Hoeveel Fte bent u werkzaam?
2. Hoe beschouwt u uw kennis over
   Evidence-Based Medicine (EBM) - Matig
    - Gemiddeld
    - Goed
    - Zeer goed
3. Hoe belangrijk vindt u het om zelf te werken
    volgens principes van EBM?

Zeer onbelangrijk

Onbelangrijk

Neutraal

Belangrijk

Zeer belangrijk

1. Begeleidt u arts-assistenten? Ja / Nee
2. Begeleidt u coassistenten? Ja / Nee

**Deel 2.**

Wij willen u vragen naar uw huidige standpunten ten aanzien van Evidence-Based Medicine (EBM). Wilt u een kruisje zetten op een punt in de lijn die overeenkomt met uw mening of antwoord, bijvoorbeeld: "Bij kinderen met een otitis media en koorts schrijf ik altijd een antibioticum voor."

|  |  |  |  |
| --- | --- | --- | --- |
|  |  |  |  |

0% 25% 50% 75% 100%

Dit antwoord geeft aan dat u het voor ongeveer 60% eens bent met de stelling.

1. Hoe zou u uw houding ten aanzien van de huidige manier waarop Evidence-Based Medicine gepromoot wordt beschrijven?

Wilt u de aanwijzer zetten op een punt in de lijn die overeenkomt met uw mening of antwoord, waarbij:
0 = zeer positief
100 = zeer negatief

|  |  |  |  |
| --- | --- | --- | --- |
|  |  |  |  |

0% 25% 50% 75% 100%

1. Hoe zou u de houding van de meeste van uw collega KNO artsen ten aanzien van Evidence-Based Medicine beschrijven ?

Wilt u de aanwijzer zetten op een punt in de lijn die overeenkomt met uw mening of antwoord, waarbij:
0 = zeer positief
100 = zeer negatief

|  |  |  |  |
| --- | --- | --- | --- |
|  |  |  |  |

0% 25% 50% 75% 100%

1. Hoe bruikbaar zijn onderzoeksbevindingen bij uw dagelijkse behandeling van patiënten?

Wilt u de aanwijzer zetten op een punt in de lijn die overeenkomt met uw mening of antwoord, waarbij:
0 = zeer bruikbaar
100 = zeer onbruikbaar

|  |  |  |  |
| --- | --- | --- | --- |
|  |  |  |  |

0% 25% 50% 75% 100%

1. Welk percentage van uw klinisch handelen vindt u op dit moment evidence-based?

|  |  |  |  |
| --- | --- | --- | --- |
|  |  |  |  |

0% 25% 50% 75% 100%

1. Het toepassen van Evidence-Based Medicine verbetert de patiëntenzorg.

Wilt u de aanwijzer zetten op een punt in de lijn die overeenkomt met uw mening of antwoord, waarbij:
0 = geheel mee eens
100 = geheel mee oneens

|  |  |  |  |
| --- | --- | --- | --- |
|  |  |  |  |

0% 25% 50% 75% 100%

1. Evidence-Based Medicine is van beperkte waarde in de KNO, omdat voor veel handelen een wetenschappelijke basis ontbreekt.

Wilt u de aanwijzer zetten op een punt in de lijn die overeenkomt met uw mening of antwoord, waarbij:
0 = geheel mee eens
100 = geheel mee oneens

|  |  |  |  |
| --- | --- | --- | --- |
|  |  |  |  |

0% 25% 50% 75% 100%

1. Het toepassen van EBM stelt, hoewel het een waardevol ideaal is, extra eisen aan de al druk bezette KNO arts.

Wilt u de aanwijzer zetten op een punt in de lijn die overeenkomt met uw mening of antwoord, waarbij:
0 = geheel mee eens
100 = geheel mee onees

|  |  |  |  |
| --- | --- | --- | --- |
|  |  |  |  |

0% 25% 50% 75% 100%

**Deel 3.**

1. Geef aan in welke mate u het eens bent met onderstaande stellingen:

|  | Helemaal oneens | Oneens | Neutraal | Eens | Helemaal eens |
| --- | --- | --- | --- | --- | --- |
| 1. Door onvoldoende ervaring met één (of meerdere) van de stappen van EBM, lukt het mij niet EBM in de praktijk toe te passen. |  |  |  |  |  |
| 2. Door tekort aan onderwijs in het toepassen van EBM, weet ik niet wat deze toepassing precies inhoudt. |  |  |  |  |  |
| 3. Ik ben niet gemotiveerd om volgens de principes van EBM te werken. |  |  |  |  |  |
| 4. Mijn vaardigheden in het zoeken naar evidence in databases (bv PubMed) zijn voldoende. |  |  |  |  |  |
| 5. Ik heb geen interesse in het zoeken naar het beste bewijsmateriaal. |  |  |  |  |  |
| 6. Het uitvoeren van een zoekactie naar klinisch bewijsmateriaal kost mij moeite. |  |  |  |  |  |
| 7. Ik zoek niet naar klinisch bewijsmateriaal omdat ik erop vertrouw dat de KNO-richtlijnen mij al voorzien van de juiste kennis. |  |  |  |  |  |
| 8. Ik heb moeite met de Engelse taal waarin onderzoek geschreven is. |  |  |  |  |  |
| 9. Als ik zoek naar literatuur weet ik niet wanneer ik tevreden kan zijn met het gevonden antwoord. |  |  |  |  |  |
| 10 Als ik een klinische vraag heb, neem ik zelf het initiatief een evidence-based antwoord te zoeken. |  |  |  |  |  |
| 11. In tijden van drukte leg ik geen prioriteit bij het zoeken naar klinisch bewijsmateriaal. |  |  |  |  |  |
| 12. Ik waardeer het als collega’s met mij nieuwe evidence overleggen. |  |  |  |  |  |
| 13. Het kritisch beoordelen van literatuur (critical appraisal) kost mij geen moeite. |  |  |  |  |  |
| 14. Bij het beantwoorden van klinische vragen, kies ik liever voor een snelle dan een precieze methode. |  |  |  |  |  |
| 15.. Tijdens een poliklinisch consult heb ik voldoende tijd om volgens de principes van Evidence-Based Medicine te werk te gaan. |  |  |  |  |  |
| 16. De tijd die ik per patiënt heb, is onvoldoende voor het zoeken naar antwoorden op mijn vragen (werkend volgens de principes van EBM)" |  |  |  |  |  |
| 17. Mijn collega KNO artsen stimuleren mij in het gebruik van Evidence-Based Medicine |  |  |  |  |  |

|  | Helemaal oneens | Oneens | Neutraal | Eens | Helemaal eens | Niet van toepassing |
| --- | --- | --- | --- | --- | --- | --- |
| 18. Mijn AIOS en coassistenten motiveren mij in het toepassen van EBM. |  |  |  |  |  |  |

1. a) Hoe vaak heeft u (of iemand voor u) de afgelopen maand een zoekactie gedaan naar wetenschappelijke literatuur?

b) In hoeveel procent van deze gevallen heeft het uw klinische handelen beïnvloedt?

1. Hebt u ooit een training gevolgd in EBM?
2. Waar hebt u toegang tot full-text bestanden in Medline/PubMed (of andere bibliografische databases)?

- Thuis
- Op mijn werkplek, in de spreekkamer
- Op mijn werkplek, buiten de spreekkamer
- Elders, namelijk:
- Nergens

1. Er zijn naast de KNO-richtlijnen een groeiend aantal tijdschriften, review publicaties en databases die relevant zijn voor EBM. Wilt u aangeven in welke mate u bekend bent met deze bronnen en of u deze gebruikt? (meerdere vakken kunnen worden aangekruist)

| Bron | Onbekend | Ik ben op de hoogte van het bestaan, maar ik gebruik het niet. | Ik weet hoe ik het moet vinden, maar ik gebruik het niet. | Ik gebruik het bij mijn klinische besluitvorming | | | |
| --- | --- | --- | --- | --- | --- | --- | --- |
|  |  |  |  | Zelden (<1x/mnd) | Soms  (± 1x/mnd) | Vaak (wekelijks) | Altijd  (dagelijks) |
|  | | | | | | | |
| KNO tijdschrift |  |  |  |  |  |  |  |
| NTvG |  |  |  |  |  |  |  |
| Pubmed / Embase |  |  |  |  |  |  |  |
| Richtlijnen (KNO/CBO) |  |  |  |  |  |  |  |
| Google |  |  |  |  |  |  |  |
| Cochrane |  |  |  |  |  |  |  |
| Uptodate |  |  |  |  |  |  |  |

1. Heeft u de afgelopen twee weken oorspronkelijke (primaire) studies opgezocht? (Zo ja, ga naar vraag 7, zo nee ga naar vraag 8)
2. Als u de afgelopen twee weken oorspronkelijke (primaire) studies heeft opzocht, in hoeverre las en beoordeelde u deze?

oit

No

ak

Va

ms

So

Zelden

A

ltijd

Lezen delen artikel

Lezen abstract

Beoordelen methodologie

Lezen gehele artikel

Beoordelen toepasbaarheid

Beoordelen validiteit

Beoordelen relevantie

1. In hoeverre hebben onderstaande factoren de afgelopen twee weken bijgedragen aan uw uiteindelijke klinische besluitvorming?

Vaak

Soms

Zelden

oit

No

A

ltijd

Mijn intuïtie/gevoel

Gevonden bewijsmateriaal

Mijn voorkeur als arts

De voorkeur van de patiënt

De voorkeur van mijn collega

De toestand van de patiënt

De prognose van de patiënt

De mening van een andere specialist

De mening van mijn aios (indien u KNO arts bent)
